# Ovarian myxoid leiomyosarcoma on PET/CT and enhanced CT: A case report and literature review

**DOI:** 10.22038/aojnmb.2026.93894.1694

**Published:** 2026

**Authors:** Ling Wang, Peng Sun, Kun Jiang, Yongfeng Liu, Youling Li, Changjun Zhao, Lei Luo

**Affiliations:** Department of Nuclear Medicine, Renmin Hospital, Hubei University of Medicine, Hubei, China

**Keywords:** Myxoid Leiomyosarcoma(MLMS) Ovarian, Positron Emission Tomography (PET), Computed Tomography (CT)

## Abstract

Primary leiomyosarcoma of the ovary is extremely rare, accounting for only 1% of ovarian tumors. Myxoid leiomyosarcoma (MLMS) is one of the subtypes. As far as we know, only few cases have been reported, and there is no imaging report of ovarian MLMS. This paper reports a case of primary MLMS of the ovary with multiple complex predominantly cystic masses and mild metabolism on PET/CT, and reviews the literature.

A 70-year-old woman was admitted to the hospital due to intermittent abdominal distension accompanied by edema of lower limbs for 3 months. Abdominal CT showed multiple irregular complex predominantly cystic masses in the abdominal and pelvic cavity, with unclear lesion boundaries, and patchy mild to moderate enhancement. PET/CT showed inhomogeneous low uptake of FDG, SUV_max_: 2.2；In the cystic area, there are multiple flocculent flotations with no FDG uptake. The patient underwent tumor debulking and omentectomy. Intraoperative exploration revealed that the tumors were complex, predominantly cystic masses accompanied by multiple solid septa, which originated in the right ovary. Finally, ovarian MLMS was histologically confirmed.

Due to extensive myxoid degeneration, MLMS of the ovary is mostly manifested as large complex predominantly cystic masses on imaging, with uneven and mild uptake of glucose. In this paper, enhanced CT and FDG PET/CT findings of ovarian MLMS were reported, which made up the gap in the imaging of this disease.

## Introduction

 Sometimes leiomyosarcoma may be associated with extensive myxoid degeneration (＞50%), so the gross specimen is gelatinous, and a large amount of myxoid substance can be seen between tumor cells under microscope, which is called myxoid leiomyosarcoma (MLMS) (1). MLMS is a very rare mesenchymal malignancy, usually occurring in the uterus (2). Myxoid leiomyosarcomas originating in the ovary are quite unusual and were first reported by Nogales et al in 1991 (3). To date, there are only six cases have been reported. Its clinical manifestations are similar to typical ovarian leiomyosarcoma, with no obvious special clinical symptoms in the early stage. As the tumor grows, abdominal pain, abdominal distension, and other peripheral tissue compression symptoms will appear. We have not found any imaging report about myxoid leiomyosarcoma of the ovary until now. Therefore, here we report a case of ovarian MLMS with multiple cystic low-density shadows in the abdomen and pelvis on FDG PET/CT, and mild FDG uptake (maximum standardized uptake value (SUV_max_): 2.2). The lesions showed patchy mild to moderate enhancement on enhanced CT.

## Case presentation

**Figure 1:**
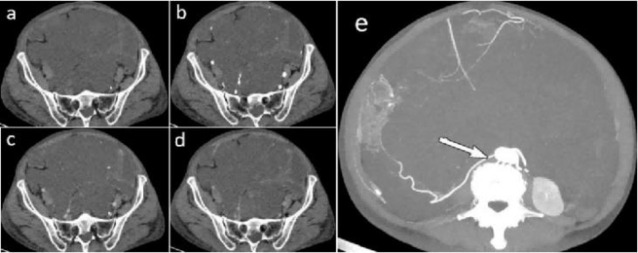
Axial plain (**a**) and contrast CT (**b**: arterial phase, **c**: venous phase, **d**: delayed phase) demonstrates multiple irregular low-density shadows in abdomen pelvic cavity, with arterial blood supply and local mild to moderate enhancement. The abdominal CT angiography (**e**) demonstrated that the tumor had an abundant blood supply

**Figure.2:**
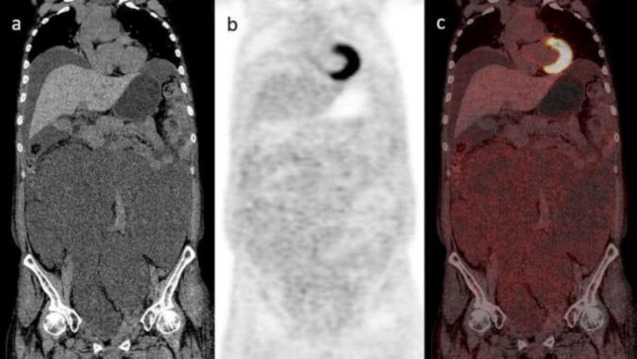
The coronal CT (a), PET (b), and fused (c) images showed multiple low-density masses of different sizes in abdomen pelvic cavity, and mild increased FDG uptake with SUV_max_ of 2.2

 A 70-year-old woman was admitted to the hospital due to intermittent abdominal distension accompanied by edema of lower limbs for 3 months. A hysterectomy for uterine fibroids was performed 15 years ago. Physical examination revealed a suspicious positive shifting dullness and pitting edema of lower extremities. Blood routine examination showed no obvious abnormality. Serum levels of alpha-fetoprotein (AFP), carcinoembryonic antigen (CEA) and carbohydrate antigen 19-9 (CA19-9) were all within normal limits, while cancer antigen 125 (CA-125) was elevated at 107.3U/mL (normal range 0-35.0). Abdominal ultrasound suggested abdominal effusion. On plain CT scan (Figure 1), complex predominantly cystic masses were seen in the abdomen and pelvis. The largest one was about 35.7×14.8×25.5cm in size, with an average CT value of 16HU and unclear lesion boundaries. Bilateral ovarian artery branches were observed after enhancement, and patchy mild to moderate enhancement was observed locally. Therefore, enhanced CT considered neoplastic lesions may be originating from the ovaries. For preoperative evaluation, the patient underwent systemic FDG PET/CT examination (Figure 2). Complex predominantly cystic masses accompanied by multiple solid septa were observed in the abdomen and pelvis on PET/CT, with a low FDG uptake with SUV_max_ of 2.2；In the cystic area, there are multiple flocculent flotations with no FDG uptake. Besides, multiple hydrops were found around the liver and spleen and between the intestines, the bladder was displaced under pressure.

The patient underwent tumor debulking and omentectomy. Intraoperative exploration revealed that the tumors were complex, predominantly cystic masses accompanied by multiple solid septa, which originated in the right ovary. The surface of the tumor adhered extensively to the pelvic wall, omentum, bowel, bladder and right ureter, and invaded retroperitoneum and right pelvic vessels. A total of about 10 kg of rotten fish meat tissue was removed. Cut surface showed an ill-defined gelatinous mass with areas of necrosis.

**Figure 3:**
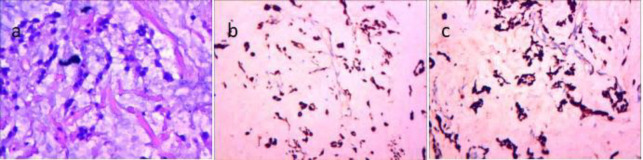
Microscopic examination (**a**; Hematoxylin-eosin stain; original magnification: ×400) showed in a mucous background, neoplastic spindle cell proliferation arranged interlacing bundles with hyperchromatic nuclei. Immunohistochemically, the tumor cells were strongly and diffusely positive for vimentin (**b**; Original magnification, ×200) and desmin (**c**; Original magnification, ×200), negative for smooth muscle actin, cytokeratin

Microscopic examination (Figure 3a) showed in a mucous background, neoplastic spindle cell proliferation arranged interlacing bundles with hyperchromatic nuclei. Our case had a low mitotic count of 2 MF（mitotic Figures）/10HPF，and the Ki-67 staining showed that the proportion of the positive tumor cells were approximately 30%. Immunohistochemically, the tumor cells were strongly and diffusely positive for vimentin (Figure 3b; original magnification, ×200) and desmin(Figure 3c;original magnification,×200)， negative for smooth muscle actin, cytokeratin. In addition, estrogen and progesterone receptors were also positive. Thus, the final histological diagnosis was MLMS of the ovary.

## Review of the literature

 We searched the PubMed /MEDLINE and Google scholar database for previous cases published before August 2020, with the terms "myxoid ", "leiomyosarcoma or smooth muscle sarcoma " and "ovary". A total of 4 English literatures (3-6) were retrieved, including 6 cases of myxoid leiomyosarcoma of the ovary. The conventional clinicopathologic features of these patients are summarized in Table 1. 

**Table 1:** Reported Cases of Primary Ovary Myxoid Leiomyosarcoma

**Reference**	**Age**	**Location**	**Size (cm)**	**Stage**	**Mitoses**	**V** **imentin**	**D** **esmin**	**SMA**	**CK**	**Follow-up**
Case 1 (3)	32	unilateral	12×9×9	I	0-1	+	*US	+	-	*LFOD 36mo
Case 2 (3)	46	unilateral	20×20	III	0-2	+	*US	+	-	*DOD 24 mo
Case 3 (3)	68	unilateral	35	III	＞20	+	*US	+	-	*DOD 13 mo
Case 4 (4)	26	unilateral	9×6×3	*US	10-12	+	*US	+	-	*US
Case 5 (5)	50	bilateral	/	/	/	/	/	+	/	/
Case 6 (6)	47	bilateral	13L/10R	IIIb	7	US	+	US	+	*DOD 3 mo
Our case	70	unilateral	35×14×25	IIIb	0-2	+	+	-	-	*LFOD 12mo

US: Un Specified; DOD: Died Of Disease; LFOD: Living Free Of Disease

## Discussion

 Leiomyosarcoma is an invasive soft tissue tumor, which most often occurs in the uterus, gastrointestinal tract, retroperitoneum and limbs. Primary ovarian sarcomas are rare, usually occurring in postmenopausal women, it has been reported to occur in less than 1% of ovarian malignancies (7). When ovarian leiomyosarcomas are associated with extensive myxoid degeneration (＞50%), it is called myxoid leiomyosarcoma（MLMS）. MLMS of the ovary is very rare and its clinical characteristics are not fully understood. The reported cases have the following clinical characteristics: 

1）Age of onset: the onset age ranges from 26 to 68 years old, with an average age of 45.1 years. Our patient is 70 years old, the oldest patient reported so far.

 2） Location of the disease: Among the previously reported cases, 4 were unilateral and 2 were bilateral; our case was unilateral. 

3）Symptoms: No special symptoms in the early stage, most patients visited the hospital because of abdominal mass and pain, by which time the patient was in an advanced stage. Our patient was in stage IIIb and accompanied by peritoneal effusion.

 Ovaries leiomyosarcoma is a nonspecific interstitial tumor, the source of its organization is unclear. There are several opinions at present:

1) The tumor originates from malignant transformation of an ovarian leiomyoma, among which the sources of ovarian smooth muscle include ovarian stromal vascular smooth muscle cells, smooth muscle cells of ovarian ligament attached at the ovary end, remnants of accessory renal tube, ovarian smooth muscle cells or interlobular tissues, and multipotent mesenchymal tissue in ovarian teratomas.

2) Migration from uterine leiomyoma, or metastasis from benign uterine leiomyoma, and eventually malignant degeneration. Similar to the report of Bouie et al. (8), our patient also had a previous history of uterine fibroids, so we believe that the occurrence of ovarian leiomyosarcoma is related to the degeneration of uterine fibroids.

 No anatomical imaging can provide accurate diagnosis preoperatively. Tsuyoshi et al. (9) thought that the ultrasonic, computerized tomography (CT) or magnetic resonance imaging (MRI) examination can clearly identify the size, shape and internal structure of the tumor, but they cannot determine the source of the tumor, let alone accurately identify the degenerative fibroids and sarcomas. 

 Preoperative PET/CT can provide more detailed functional and anatomical data for further diagnosis of patients with suspected malignant tumors. At present, we have not found imaging reports of myxoid leiomyosarcoma of the ovary. There are only a few reports on myxoid leiomyosarcoma of uterus and pleura, and most of them show huge cystic solid lobulated mass with hydrothorax or ascites. In PET/CT, tumor metabolism was moderately increased or not increased, and SUV_max_ was between: 1.9-8.2 (1). In addition, a distinctive mucoid or gelatinous gross appearance is common and usually is the first indication of the diagnosis. Size is variable, but tumors are typically large (mean, 20.6 cm). The final diagnosis of MLMS of the ovary depends on pathological examination and immunohisto-chemistry. However, due to the extensive myxoid degeneration of the tumor, the tumor cells are relatively sparse, and its histological evaluation is difficult. Previous studies (3) suggested that the solid region of the tumor and the mucus should be distinguished when the mitosis count was carried out, and the solid region mitosis count was taken as the final result. Previous studies (9) of MLMS in utero have found that patients who die usually have higher rates of mitosis. Among the three patients who died of ovarian MLMS, mitosis counts were 7/HPF and >20/HPF in two patients, 0-2/HPF in one patient. The mitosis rate in the surviving patients were <2/HPF. 

 Also, Carlos found that all cases with a histologic appearance diagnostic of MLMS expressed at least 1 smooth muscle marker. As expected, smooth muscle actin and desmin were the most sensitive. Radiologic examination of ovarian leiomyosarcoma usually shows abundant blood vessels with a typical nonvascularized central area (10). The differential diagnosis of ovarian MLMS includes the following:

① Carcinosarcoma of ovarian, it can be distinguished from MLMS due to its epithelioid component.

②Malignant fibrous histiocytoma and fibrosarcoma of the ovary: Meigs syndrome is often associated clinically. 

③Ovarian schwannoma: it has a complete capsule, and the tumor cells are thin and the nuclear palisade is arranged. Immunohisto-chemistry was positive for S-100 protein and negative for Desmin. 

④Malignant mesodermal mixed tumor of the ovary: it contains Mullerian ducts, whereas leiomyosarcomas do not. 

⑤Subserosal uterine leiomyoma with pedicle: It is a subserosal leiomyoma that attaches to the ovary and is supplied by the ovary blood vessels for its growth. This type of tumor is extremely rare and is characterized by attachment to the ovarian surface without entering the parenchyma.

 The incidence of ovarian leiomyosarcoma is low, and its treatment is given priority to with operation. There is no consensus on whether to conduct radiotherapy or chemotherapy after an operation. Tsuyo et al. (11) thought that given postoperatively in patients with ovarian leiomyosarcoma of gemcitabine and docetaxel chemotherapy can improve the tumor progression, but Tsuyoshi (12) pointed out that because sarcoma is not sensitive to radiation and chemotherapy, so it couldn't improve the prognosis of the disease. Since the patient was old and poor in physical condition, and had a greater risk of chemotherapy, our patient did not undergo postoperative chemoradiotherapy. Follow-up for 10 months, the patient was in good condition and without recurrence.
